# Poly-victimization in a Norwegian adolescent population: Prevalence, social and psychological profile, and detrimental effects

**DOI:** 10.1371/journal.pone.0189637

**Published:** 2017-12-14

**Authors:** Svein Mossige, Lihong Huang

**Affiliations:** 1 Department of Psychology, University of Oslo, Oslo, Norway; 2 NOVA – Norwegian Social Research, Oslo and Akershus University College of Applied Sciences, Oslo, Norway; University of Westminster, UNITED KINGDOM

## Abstract

**Background:**

This study focuses on poly-victimization, with the aim of providing a realistic estimation of the prevalence of lifetime victimization in a Norwegian adolescent population (ages 18–19 years).

**Methods:**

Based upon the concept from previous research, we applied measures of child poly-victimization on Norwegian data obtained from a national youth survey in 2015 (N = 4,531) to arrive at an estimation of its prevalence. We used variables that measure individual characteristics such as gender and educational aspiration and socio-economic factors such as parents’ education level and home economic situation to derive a social and psychological profile of victimization and poly-victimization among young people. Finally, we estimated the effects of poly-victimization on mental health such as symptoms of depression, anxiety and trauma.

**Results:**

Our study identified a poly-victimization prevalence of 8.6% among young people, i.e. they were exposed to three of all four forms of violence investigated by our study: non-physical violence, witnessing violence against parents, physical violence and sexual abuse. Adolescents of poly-victimization are six times more likely to report depression and anxiety and trauma when compared with those without victimization.

**Conclusions:**

Poly-victimization is a phenomenon that heavily burdens many young people across many national contexts. Poly-victims clearly tend to develop depression, anxiety, and posttraumatic stress symptoms. The early detection of sexual abuse, physical violence, and bullying victimization is of critical importance and preventive measures could consider addressing family factors through parental educational programs.

## Introduction

Children who are exposed to one type of maltreatment are often also victims of other types of violence, as different forms of victimization tend to repeat and co-occur [1–4]. Research on increased exposure to different types of childhood adversities indicates that there is a corresponding increase in the rates of mental health problems [5]. Poly-victimization is a concept proposed and conceptualized to describe the situation where a child or adolescent has been exposed to high levels of different types of victimization [6, 7].

Over the past decade, we have witnessed the development of various concepts and methodological approaches that estimate various risk factors associated with children who are victims of maltreatment and violence. Musicaro et al., [8] offers a clarification of the terminology on recurrent interpersonal victimization by designating how cumulative trauma theory emphasizes the relationship between the frequency and severity of victimization which posits a linear association between the number of types of traumatic events and the severity of clinical impairment. In the research literature, we find two concepts used to describe children and youth who have been exposed to more than one type of violence: poly-victimization and multiple victimization [9–14]. The concept of poly-victimization first emerged from a series on the Developmental Victimization Survey (DVS), which was administered to a nationally representative sample of 2,030 children aged 2–17 years living in the United States [7]. Finkelhor and associates [2, 6, 7, 15] developed several measures of poly-victimization based on their ability to predict trauma symptoms, which represent one of the most important correlates of, and reasons for, identifying poly-victimization. Poly-victimization was originally defined as “the experiencing of four or more different types of victimization in different incidents in a given year” [6, 7] which includes children who have experienced victimization levels above the mean number of 3.0 victimizations identified by the survey. Eventually, Finkelhor and colleagues [15] proposed identifying poly-victimization as involving the 10% of children who experienced the highest numbers of different types of victimizations, such as sexual abuse, physical abuse, bullying, and exposure to family violence. By this definition, they emphasize that measures of poly-victimization should include different kinds of victimization instead of multiple episodes of the same kind of victimization [15]. This is in accordance with the definition that we apply in this study.

### Measures of prevalence in previous research

The use of the term poly-victimization has quickly evolved and it now distinguishes between childhood poly-victimization, lifetime poly-victimization, and recent (in the past year) poly-victimization. In the United States, the findings of the DVS (N = 2,030; respondents were between the ages of 2–17 years) estimated that the prevalence of poly-victimization (i.e. those who experienced at least four types of victimization) was 22% among children aged 2–17 years. Of these respondents, 15% experienced low poly-victimization (four to six types of victimization) and 7% experienced high poly-victimization (seven or more types of victimization)[7]. Meanwhile, another study conducted in the US (the NatSCEV II study) estimated that 17.8% of children and adolescents between the ages of 10–17 years belonged to a latent group of poly-victims who had been victimized in multiple settings, by different perpetrators, in incidents involving weapons and sexual violence [4].

Several prevalence studies of poly-victimization have been conducted in other countries using representative samples and substantial adaptation of the Juvenile Victimization Questionnaire (JVQ) [7], e.g. in the United Kingdom [16], in Spain [17], in Canada [18], in Sweden [19]. We observe in the literature a lack of consensus in terms of both the measurement of poly-victimization and the ‘cutoff’ point that determines who can qualify as a poly-victim. There are a variety of definitions and measurements used with respect to the term poly-victimization. Some researchers choose to use a specific number of victimization types e.g. [6, 17] to construct the concept of poly-victimization while others take into account victimization contexts [e.g. 4] or numbers of events [e.g. 19]. Moreover, various study results show an accumulation of poly-victimization that occurs from childhood to adulthood and in these instances, the numbers and types of victimization increase in association with age.

Nonetheless, there is the claim of a strong linear relationship between poly-victimization and mental health over both the short term and long term [20]. Children are negatively affected in many ways by experiences of maltreatment, abuse, and neglect [21]. This study will apply the concept and previous measures of poly-victimization with a Norwegian adolescent population sample. We will test our hypothesis that the same linear association between poly-victimization and mental health also can be found in the Norwegian context.

## Data and methods

### Procedure

A national youth survey conducted in spring 2015 in Norway, known as UngVold 2015 study, was designed to assess the prevalence of offences and violence committed against children and adolescents [22]. This survey used a stratified random sample of all third-year students in 49 schools. All third-year students were invited to complete a questionnaire on an individual computer during school hours with a teacher present. Complying with the Personal Data Act (Act of 14 April 2000 No. 31 relating to the processing of personal data) § 33 and § 34, this research was approved by the Norwegian Data Protection Authority (NSD) 14/01407-5/EOL before data collection. Then we contacted sampled schools to obtain permission at the school level for conducting the survey. Students at each school then gave their consent to participate in the survey after receiving clear written information of the themes and purpose of the study. In both written information to schools and to individual students, it was emphasized that it was voluntary to participate and all answers would be anonymous. The anonymity of the survey was further safeguarded both at school and individual levels in a way that we did not request any direct or indirect person-identification data.

As the topics in the questionnaire were sensitive and could awaken unpleasant memories or feelings in the respondents, we provided information about where and whom (counselor or health sister at school) the respondents could seek help from, both at the start of the survey and at the last page of the survey questionnaire.

### Participants

Of the 6,848 students invited, 4,530 students completed the survey, which yielded a sufficient response rate of 66%. A few factors contributed to this response rate: 1) students could refuse to participate in the study, as per Norwegian research ethic regulations; 18-year-old students can determine whether they wish to consent to participate in research; 2) unfortunately, some of the students may not have been at school during the UngVold 2015 data collection period. This latter point was especially true for male students, who were mostly completing field practice during their last year of upper-secondary education.

After conducting some preliminary data analyses, we did not observe any systematically missing data from the Ungvold 2015 survey. Most of the respondents (93.7%) were 18–19 years of age at the time of the survey and 59% were female. Nevertheless, our data featured some limitations with regard to our ability to estimate the prevalence rate of victimization and the representativeness of the Norwegian youth in this age group. As this was an upper-secondary school based survey, the sample under-represented (or, unfortunately, excluded) two groups of young people. First, approximately 2% of Norwegian young people do not attend upper-secondary school education upon completion of compulsory education. Second, approximately 30% of Norwegian upper-secondary school students drop out of school between the first and last years of their education [23].

### Measures of non-physical, witness of domestic violence, physical, and sexual abuse

The UngVold 2015 survey obtained data on four major forms of offences against children: 1) non-physical violence (severe verbal bullying, threat of violence) perpetrated by parents and/or peers; 2) witnessing non-physical and physical violence against their parents by parents’ partners; 3) physical violence (slap with open hands, fists, or being “beaten up”) perpetrated by parents or peers; and 4) sexual abuse (unwanted touching, exposure, or sexual acts) perpetrated by adults in or out the family and/or by peers. The study differentiated between offences committed by adults and those by peers both before and/or after the student reached the age of 13–14 years. Table 1 presents all of the items and their response alternatives, which measured victimization of violence in four forms used in the UngVold 2015 survey.

**Table 1:** Measures of victimization based on the four forms of violence and abuse by perpetrators.

			Childhood (before/at the age of 13/14 years)	Adolescence (after the age of 13/14 years)
Forms of abuse	Category perpetrators	Measured items	Response alternative	Response alternative
Non-physical abuses	By peers (including siblings)	1. Been seriously troubled or threatened or excluded by peers 2. Been sexually harassed by peers 3. Been threatened with violence by peers	0 = never 1 = once 2 = a few times 3 = monthly 4 = weekly 5 = daily	0 = never 1 = once 2 = a few times 3 = monthly 4 = weekly 5 = daily
	By Parents*	1.Had your mother/father creamed at, humiliated, or thrown/hit/kicked something toward you? 2.Had your mother/father threatened you with violence?		
Witness violence	Witness parents being abused*	1. Seen or heard mother/father being shouted at 2. Seen or heard mother/father being insulted or humiliated 3. Seen or heard mother/father being threatened with violence 4. Seen or heard mother/father being pushed or heavily shaken 5. Seen or heard mother/father having hair pulled or pinched 6. Seen or heard mother/father being slapped with an open hand 7. Seen or heard mother/father being hit with a fist 8. Seen or heard mother/father being beaten with an object 9. Seen or heard mother/father being seriously beaten up 10. Seen or heard mother/father being subjected to other violence		
Physical abuse	By peers (including siblings)	1. Been beaten without visible injury by peers 2. Been injured by violence perpetrated by peers without needing doctoral treatment 3. Been injured by violence perpetrated by peers that required doctoral treatment		
	By parents*	1. Had mother/father pushed or heavily shaken you during an argument with you? 2. Had mother/father pulled your hair or pinched you during an argument with you? 3. Had mother/father slapped you with an open hand during an argument with you? 4. Had mother/father hit you with a fist during an argument with you? 5. Had mother/father beaten you with an object during an argument with you? 6. Had mother/father really beat you up during an argument with you? 7. Had mother/father performed other acts of violence toward you during an argument with you? 8. Had you been injured as a result of violence by mother/father?		
Unwanted (against your will or by force) sexual events	By adults or peers at home, school, or strangers	1. Have someone touched your genitals? 2. Have you touched your sex in front of someone? 3. Have you touched someone’s sex? 4. Have you masturbated in front of someone? 5. Have you had intercourse? 6. Have you had oral sex? 7. Have you had anal sex? 8. Have you had other forms of sex? 9. Has anyone tried to rape you? 10. Have you been raped?	0 = never 1 = once 2 = a few times	0 = never 1 = once 2 = a few times

Note:

*Violence experienced at home (parents) has a response alternative for those younger than 14 years old and those older than 14 years old.

Measures of non-physical abuse include the following sub-categories: 1) verbal bullying and threats of violence perpetrated by peers overall, before and after the age of 13 years; and 2) verbal bullying and threats of violence perpetrated by parents. Witness violence includes witnessing verbal abuse between parents and witnessing physical violence between parents overall, before and after the age of 14 years. Measures of physical violence include 1) being beaten or injured by peers, siblings, or other young strangers; and 2) physical abuse perpetrated by parents overall, before and after the age of 14 years. Measures of sexual abuse include items of unwanted sexual events overall, before and after the age of 13 years. These instances of abuse were captured in a question asking students if they had experienced any of these events against their wills.

### Analysis plan

We analyzed measures of victimization using the framework of four forms of violence—i.e., non-physical violence (verbal or psychological abuse), witnessing violence against parents, physical violence, and sexual abuse. We first identified a poly-victimization group that experienced all four forms of violence by counting all instances where the respondents chose the response options ‘once’ or ‘more times’ on those items that measured physical and sexual abuse, while excluding items measuring non-physical/verbal abuse that happened only ‘once’. We then looked into compositions of poly-victimization that were most commonly experienced during each life phase (childhood or adolescence) in our group. Furthermore, we provided a social and psychological profile of the adolescents who were exposed to violence and poly-victimization.

Indicators for the adolescents’ social profile included individual characteristics such as gender, country of birth, school achievement, and educational aspirations, as well as home background variables such as parents’ education and employment, whether or not the parents were living together, and home economic situation. Home economic situation was a subjective measure that was captured by asking ‘Has your family had a good or bad economic situation in the past two years?’ on a six-point scale, with responses ranging from ‘1’ (a lot ups and downs), ‘2’ (bad economy all the time), ‘3’ (bad economy most of the time), ‘4’ (neither good or bad), ‘5’ (good economy most of the time), and ‘6’ (good economy all of the time). We combined ratings of ‘2’ and ‘3’ to create a group variable termed as ‘Home has bad economy’ which was coded as ‘1’ and all other scenarios were coded as ‘0’.

A full version of the Hopkins Symptom Checklist (HSCL) [24], which features 26 items, served as an indicator of the young people’s mental health profiles. The internal consistency of these subscales has appeared to be good and Cronbach´s alpha range between 0.81 and 0.92 for depression, and between 0.82 and 0.87 for anxiety in population samples in Norway, Denmark, Finland and Germany [25]. The 26-item HSCL measures the level of depression and anxiety experienced over the last week on a four-point scale, with responses including 1 = ‘not troubled at all’, 2 = ‘a little troubled’, 3 = ‘troubled quite a lot’, and 4 = ‘very much troubled’. The depression and anxiety variable is an average score that is obtained from the sum of the 26 HSCL items (mean = 1.66, Std = 0.57). Higher values indicate poorer mental health.

A short screening scale was used to measure posttraumatic stress disorder (PTSD) [26]. The short screening scale for PTSD is a seven-item checklist that features ‘tick-box’ responses indicating whether the respondent had experienced certain symptoms during the past month. A score of 4 or greater on this scale defined positive cases of PTSD with a sensitivity of 80%, specificity of 97%, positive predictive value of 71%, and negative predictive value of 98%. A PTSD score is constructed by adding all of the items that had been selected; responses ranged from 1–7 (mean = 0.74, Std = 1.50).

Finally, we used logistic analysis technique to estimate the effect of poly-victimization on the mental health of young people. We consider regression coefficients produced by linear regression analysis are difficult to interpret since most of the variables used in the study are categorical. For the sake of clear interpretation in a pedagogical way, we choose to use logistic regression analysis as it can provide odds ratio (OR) of each increase of the types of victimization on young peoples’ ‘membership’ into poor mental health. We constructed a ‘poor mental health’ group of young people, as indicated by the HSCL value; moreover, a PTSD value ≥2 is higher than the whole group means by 0.5 standard deviation (i.e., 1.90 for HSCL and 1.51 for PTSD). Moreover, we consider a value of 2 in HSCL to be appropriate, as it represents the response alternative ‘2 = ‘a little troubled’ on all HSCL symptom items while a value of 2 corresponds to two symptoms listed in DSM-IV.

## Analysis and results

### Prevalence of victimization types

Table 2 presents the prevalence of all types of victimization that occurred during two phases of the young people’s lives—i.e., victimization during childhood (before the age of 13 or 14 years) and adolescence (at and after the age of 14 years)–reported by the same participants.

**Table 2:** Prevalence of the different types of victimization, in terms of the four forms of violence and across 12 situations (denoted as s1-s12)*, and the compositions of poly-victimization (%).

Forms, types and situations of victimization		Any victimization (n = 2829) (% of N)	Poly-victims’ experience of each type of victimization (% of n)		
			Single form (n = 1139)	Two forms (n = 899)	Poly-victimization (n = 795)
Total N = 4531		62.4	25.1	19.8	17.6
Non- physical violence	By peers, overall	40.5	43.5	74.5	84.4
	(s1) By peers before the age of 13 years	36.2	38.4	66.3	76.5
	(s2) By peers after the age of 13 years	25.6	23.2	44.8	62.0
	By peers both before and after the age of 13 years	21.3	18.0	36.6	54.1
	(s3) By parents#	10.8	1.9	12.0	45.3
Witness violence	(s4) Witnessed verbal abuse between parents#	24.9	18.6	33.3	77.5
	Witnessed parents being physically abused, overall	7.9	3.7	7.1	31.4
	(s5) Witness before and at the age of 13 years	6.3	2.3	5.2	26.5
	(s6) Witness at and after the age of 14 years	4	1.3	3.6	16.7
	Witness both before and after The ages of 13–14 years	2.4	0.1	1.7	11.9
Physical violence	By peers, overall	18.8	8.9	37.5	51.8
	(s7) By peers before the age of 13 years	15.2	6.0	30.7	43.1
	(s8) By peers after the age of 13 years	10.3	4.7	19.1	30.4
	By peers both before and after the age of 13 years	6.7	1.8	12.3	21.8
	By parents, overall	20.6	11.7	29.5	67.5
	(s9) By parents before and at the age of 13 years	16.5	9.2	20.1	58.2
	(s10) By parents at and after the age of 14 years	13.7	5.6	19.2	48.2
	By parents both before and after the ages of 13–14 years	10.0	3.6	10.9	39.6
Sexual abuse	Sexual abuse, overall	19.6	15.8	27.5	58.2
	(s11) Sexual abuse before the age of 13 years	6.0	3.7	7.3	20.9
	sS12) Sexual abuse after the age of 13 years	17.3	13.6	24.5	51.6
	Sexual abuse both before and after the age of 13 years	3.7	1.5	4.3	14.2
Total account of 12 situations, mean (std.)		1.87 (2.21)	1.28 (0.48)	2.86 (0.98)	5.57 (1.89)

Note:

* (s1)–(s12) each denotes one situation of violence;

^#^ few respondents provided the age at which they witnessed violence between their parents or when they were exposed to verbal abuse perpetrated by their parents.

Our measures show that 62.5% of young people in Norway have experienced any form of victimization during their lifetime up to the ages of 18–19 years. Our analysis identified that 8.6% of young people in the group experienced poly-victimization (i.e., victimization of all four forms of violence, as investigated by the UngVold study). Meanwhile, one quarter of all young people (25%) were exposed to two forms violence and nearly one third (29%) of Norwegian adolescents were exposed to a single form of violence during their lives as children and adolescents. When examining individual categories, the highest prevalence was noted for victimization of verbal bullying perpetrated by peers (40.5%); most of these cases occurred before the age of 13 years (36.2%). Following this, substantial proportions of youth witnessed verbal abuse between parents (24.9%); moreover, they experienced physical violence perpetrated by parents (20.6%) and by peers (18.8%), and they also experienced sexual abuse (19.6%).

From the analyses presented in Table 2, we observe that the prevalence of victimization in all forms and types tends to decrease from childhood to adolescence, with the exception of sexual abuse. For example, verbal bullying by peers decreased from 36.2% before the age of 13 years to 25.6% after the age of 13 years, while physical abuse by peers decreased from 15.2% to 10.3% during the same periods. Moreover, we also observed that there was an overlap in victimization during childhood and adolescence, which indicates the continuation of victimization from childhood into adolescence for some young people. For example, 21.3% of young people were verbally bullied by their peers during both childhood and adolescence while 3.7% of young people were exposed to sexual abuse both during childhood and adolescence.

### Estimations of the prevalence of poly-victimization

As the UngVold data measure four forms of violence perpetrated by adults and peers during the two life phases of young people (childhood and adolescence), we identified 12 situations of victimization (see s1 to s12 in Table 2, first column from the left) by counting, for example, non-physical violence perpetrated by peers before the age of 13 years as one situation of victimization (s1), and any other victimization events that occurred after the age of 13 years as another situation of victimization (s2). Physical abuse perpetrated by parents up until the age of 13 years was counted as one situation of victimization (s9), and that which occurred after the age of 13 years was regarded as another (s10). In this way, we arrived at a number of total victimizations that ranged from 0–12 with a mean of 1.87 (Std = 2.21).

When we applied Finkelhor et al.’s [6] definition of poly-victimization (with three or more victimization situations), which was established using the number of victimizations above the mean (i.e., 2 in our case), it had a prevalence of 19.8% in Norway. By counting victimization as exposure to violence in four forms (non-physical, witnessing, physical, and sexual), we identified that 17.6% of Norwegian adolescents were poly-victims (i.e., those who were exposed to three or all four forms of violence). Table 2 also presents the composition of poly-victimization by showing the proportions of each type and each situation of victimization included in this definition. It is evident that each victimization types in each of the situations investigated in our study had disproportionately contributed to the poly-victimization group. For example, 36.2% of youths were exposed to non-physical violence perpetrated by peers before the age of 13 years, while 76.5% of the poly-victims had experienced this situation of victimization. Moreover, 13.7% of the young people were exposed to physical violence that was perpetrated by their parents during adolescence (and after the age of 14 years), while three times more (48.2%) poly-victims had experienced this situation of victimization. Finally, 6% of Norwegian youth were exposed to sexual abuse during childhood, while among those who had experienced poly-victimization 20.9% had been exposed to sexual abuse.

Several types and situations of victimization account for the largest proportions of poly-victimization; for instance, most respondents who experienced victimization faced peer verbal bullying (84.4% in total) during childhood (76.5%) and adolescent (62%), followed by witnessing parents been verbally abused (77.5%), and physical abuse perpetrated by parents (67.5% in total) during childhood (58.2%) and adolescence (48.2%). Eventually, our analyses showed that both calculation methods yielded rather similar estimations of poly-victimization (see the last row at the bottom of Table 2) in cases where poly-victimization included all four forms of violence; there was an average number of five types and situations of victimization (mean = 5.57, Std = 1.89), which was referred to as ‘high poly-victimization’. Meanwhile, the group that experienced two forms of violence (second column from the right in Table 2) also experienced between 2–4 situations of victimization overall (mean = 2.86, Std = 0.98), which we can refer to as ‘low poly-victimization’.

### The social and psychological profiles of poly-victimization

Table 3 presents the social and psychological profiles of all victimization groups, including the poly-victimization group identified by our study.

**Table 3:** A social and psychological profile of Norwegian youth exposed to violence and poly-victimization (percent).

	Non-victimization	Victimization of a single form of violence	Victimization of two forms of violence	Poly-victimization
N (%)	1698 (37.5)	1139 (25.1)	899 (19.8)	795 (17.6)
*Individual characteristics*				
Female*	55.5	60.7	56.6	67,9
Born in Norway	91.4	93.1	93.5	93.5
Mean (std.) school achievement in Norwegian, Mathematics and English	4.04 (0.82)	4.11 (0.79)	4.07 (0.83)	3.90 (0.82)
Mean (std.) of depression and anxiety symptoms*	1.42 (0.44)	1.61 (0.48)	1.78 (0.59)	2.07 (0.63)
Mean (std.) of posttraumatic symptoms*	0.31 (0.92)	0.58 (1.30)	0.94 (1.62)	1.66 (2.08)
Aspire to achieve higher education	75.1	76.4	75.9	73.6
*Home background characteristics*				
Father born in Norway	86.3	88.0	88.9	87.4
Mother born in Norway	86.1	89.7	89.1	87.3
Parents live together*	71.2	66.9	62.0	48.3
Father with higher education*	44.6	45.4	46.6	38.5
Mother with higher education	56.2	56.0	56.3	52.1
Father not working*	12.6	14.0	14.6	22.6
Mother not working*	14.5	12.7	16.8	23.3
Father with substance abuse*	6.8	9.3	15.1	25.8
Mother with substance abuse*	4.0	3.6	5.9	11.1
Home has a bad economic situation*	18.8	21.5	28.0	43.3
Family once lived in welfare housing*	6.1	6.1	5.6	10.2

Note: 65 of the cases had missing responses for gender (1.4%). For percentage differences between the groups, we performed Chi-squared test while for mean differences between groups, we performed independent t-test.

* indicates that the difference between ‘poly-victimization’ and ‘non-victimization’ is statistically significant at the 0.05 level.

When compared with youth who were never exposed to any form of violence or abuse (second column in Table 3), young people who experienced poly-victimization were overrepresented by girls (67.9% versus 55.5% in the non-victimization group), and by those whose families lived in houses provided by the welfare system (10.2% versus 6.1% in the non-victimization group). Young people who faced poly-victimization were also disproportionately from disadvantaged homes with bad economic situations, as perceived by the young respondents (43.3% versus 18.8% in the non-victimization group); moreover, 48.3% were living in homes where their parents were living together, while 71.3% of those in the non-victimization group had parents who were living together. In addition, fewer parents from the poly-victimization group had received higher education (38.5% of fathers and 52.1% of mothers) when compared with the parents in the non-victimization group, 44.6% of fathers and 56.2% of mothers had higher education. Moreover, more parents in the poly-victimization group were unemployed (22.6% of fathers and 23.3% of mothers), while 12.6% of fathers and 14.5% of mothers in the non-victimization group were unemployed at the time of the survey. Most importantly, young people in the poly-victimization group were substantially more likely to have parents with a substance abuse problem (25.8% of fathers and 11.1% of mothers) when compared with those in the non-victimization group with 6.8% of fathers and 4% of mothers dealing substance abuse.

Several individual and social characteristics did not exhibit significant differences between the poly-victimization group and the non-victimization group, such as respondents’ and parents’ birth countries, young people’s school achievements, and young people’s educational aspirations. However, in the poly-victimization group, we did observe a clear increase in depression and anxiety symptoms (mean = 2.07, Std = 0.63) when compared with the non-victimization group (mean = 1.42, Std = 0.44). PTSD symptoms were also greater in the poly-victimization group (mean = 1.66, Std = 2.08) than in the non-victimization group (mean = 0.31, Std = 0.92). The differences of both HSCL and PTSD between the group of poly-victimization and the non-victimization group are statistically significant (see Table 3).

### The association between poly-victimization and mental health

Table 4 presents the results of the logistic regression analysis of young people’s exposure to poly-victimization; it was found that poly-victimization predicted the youth’s recent mental health, as indicated by depression and anxiety symptoms, and by posttraumatic stress, reaching a value of 2 and higher.

**Table 4:** Logistic regression analysis of young people’s exposure to poly-victimization predicting their recent poor mental health (OR).

	Depression and anxiety ≥2 (n = 954; 21.1% of N)		Posttraumatic stress ≥2 (n = 845; 18.6% of N)	
**Background variables**	Model 1	Model 2	Model 1	Model 2
Female	4.52***	4.41***	2.24***	2.09***
Born in Norway	1.60*	1.44*	1.69**	1.48
Aspire to achieve higher education	0.82*	0.83*	0.79**	0.78*
Father born in Norway	0.98	0.95	0.89	0.86
Mother born in Norway	1.19	1.21	0.81	0.81
Parents live together	0.79**	0.90	0.73**	0.83*
Father with higher education	1.00	0.96	1.18	1.14
Mother with higher education	0.01	0.96	0.87	0.82*
Father not working	1.05	0,97	1.16	1.08
Mother not working	1.03	0.92	1.33**	1.21
Father with substance abuse	1.32***	1.18*	1.17**	1.05
Mother with substance abuse	0.99	0.95	1.09	1.04
Home has a bad economic situation	1.70***	1.38***	1.38**	1.10
Family once lived in welfare housing	1.11	1.07	1.29	1.26
**Independent variable: poly-victimization**				
Reference: Non-victimization				
Victimization of single forms of violence		2.01***		1.74***
Victimization of two forms of violence		3.80***		3.34***
Poly-victimization (three or all four forms of violence)		5.99***		5.66***
*Variance explained (Nagelkerke R Square)*	14.2%	22.6%	7.1%	15.6%
*Chi-squared / df*	415.6/14	681.6/17	195.3/14	438.9/17

Note:

*p<0.05;

**p<0.01;

***p<0.001.

As shown in Table 4, we applied two models respectively, to predict depression and anxiety, and to predict PTSD. Model 1 includes only background variables and Model 2 includes the independent variable for victimization while keeping the background variables constant.

Among all of the background variables included in the models—gender, being born in Norway, having a father with a substance abuse problem, and living in a home with a poor economic situation—all significantly increased the odds of having poor mental health. Females were four and a half times more likely than their male counterparts to have depression and anxiety symptoms (OR = 4.54), and they were twice more likely to have PTSD (OR = 2.24). Young people who were born in Norway were 60% more likely to exhibit depression and anxiety symptoms (OR = 1.60) and they were 69% more likely than those who were not born in Norway to have posttraumatic stress (OR = 1.69). Having a father with a substance abuse problem increased the odds of having depression and anxiety by 32% (OR = 1.32), and these respondents were 17% more likely to have posttraumatic stress (OR = 1.17) when the poly-victimization variable was not included in the model. Living at a home with a poor economic situation significantly increased the odds of facing poor mental health by 70% when depression and anxiety symptoms were present (OR = 1.70) and by 38% when posttraumatic stress was evident (OR = 1.38). Nevertheless, a couple of background variables did have some protective effects on the mental health of young people. For instance, aspiring to attend higher education decreased the odds of depression and anxiety by 18%, while it reduced the odds of posttraumatic stress by 21% (OR = 0.79); similarly, having a mother who attained a higher education also reduced the likelihood of developing posttraumatic stress by 18% (OR = 0.82 in Model 2).

The analysis of Model 2 for both HSCL and PTSD presented in Table 4 shows strong and significant effects of victimization on the mental health of young people. First, victimization of single forms of violence nearly doubles the odds of depression and anxiety (OR = 2.01) and posttraumatic stress (OR = 1.74). Second, victimization of two forms of violence (referred to as ‘low poly-victimization’) increased the odds of depression and anxiety by nearly four times (OR = 3.80), and the odds of experiencing posttraumatic stress increased by more than three times (OR = 3.34). Third, poly-victimization had the strongest detrimental effect on the mental health of young people, as it increased the odds of experiencing depression and anxiety symptoms by six times (OR = 5.99); it also increased the chances of developing posttraumatic stress by nearly six times (OR = 5.66).

## Discussion and conclusion

When applying the two calculation methods to identify the lifetime prevalence of poly-victimization in a young Norwegian population, both methods yielded rather similar estimates. Our study identified a poly-victimization prevalence of 8.6% among young people, i.e. they were exposed to three of and all four forms of violence investigated by our study: non-physical violence, witnessing violence against parents, physical violence and sexual abuse. This group experienced an average number of six types and situations of victimization. The prevalence of poly-victimization estimated from our sample is rather similar to those found in Sweden [19] and Canada [18], but the rate is different from those reported by a Spanish study [17]. When applying the definition of Finkelhor and associates [15], where poly-victimization should account for 10% of young people who were exposed to the highest numbers of victimization in the sample, our data indicated that this group (10% of young people) had been exposed to 5–12 types and situations of victimization.

Our study provided another piece of evidence highlighting that poly-victimization is also a phenomenon that heavily burdens many young people outside a North American context. Poly-victimization includes all types and situations of victimization investigated in our study, but certain types and situations of victimization contributed the most in this group: experiencing sexual abuse during adolescence, undergoing peer verbal bullying during childhood and adolescence, witnessing parents being verbally abused, and facing physical abuse perpetrated by parents. The association found between poly-victimization and mental health supports the idea that there is a cumulative impact of childhood adversities where increased exposure to multiple victimizing events increases the risk for various mental health problems.

Poly-victims clearly tend to develop depression, anxiety, and posttraumatic stress symptoms. Our analysis results show how those who experience victimization of three of and all four forms of violence—i.e., non-physical violence, physical violence, those who witness violence at home, and those who experience sexual abuse—are six times more likely to report depression and anxiety. Moreover, they are also six times more likely to describe having posttraumatic stress symptoms when compared with those adolescents in the non-victimization group. Our finding of a cumulative impact is in accordance with the findings of other studies [19, 27]. When compared with other young people who did not experience any victimization, the young people in the poly-victimization group identified in our study appeared to share a distinctive profile in terms of both their sociodemographic and mental health variables. Those in the poly-victimization group are overrepresented by girls; they are more likely to be from disadvantaged homes with bad economic situations, and they tend to have separated parents, unemployed parents and parents who engage in substance abuse. These individual and sociodemographic characteristics may be burdens in and of themselves, and they appear to reinforce the subsequent emergence of maladjustment.

The strong increase in the risk of developing anxiety and depression for each added form of victimization is a clear indication that the early detection of sexual abuse, physical violence, and bullying victimization is of critical importance. For every new victimization experience, individuals become increasingly more vulnerable to becoming the target for new offenders. By providing distinctive sociodemographic and psychological profiles of those young people who are more likely to experience poly-victimization, the results of our study point to some of the risk factors. In fact, the more that youth experience different types of victimizing situation, the smaller the change that these individuals will have access to a secure retreat. If a youth runs the risk of being polyvictimized preventive measures are important. Studies to identify the risks of being exposed to polyvictimization have so far indicated that many of these are to be found at a systemic level [5]. The implication of this is that implementation of preventive measures should target changes at systemic level, i.e. beyond individual factors by providing a safe environment for our young people.

### Limitations of the study

As a national youth survey, our study has data limitations with regard to the interpretations of the prevalence and consequences of the poly-victimization among Norwegian youth as it is stated in previous section “Data and Methods”. Although several measures were taken to assure the respondents both confidentiality and access to psychological counselling in answering such sensitive questions, researchers feared under-reporting due to the under-representation of specific groups and suppressed memory or memory loss of childhood abusive experiences when the respondents were asked to recall events from their childhood [22].

### Declaration

This research received full financial funding from the Norwegian Ministry of Justice and Public Security. Complying with the Personal Data Act (Act of 14 April 2000 No. 31 relating to the processing of personal data) § 33 and § 34, this research is also approved by the Norwegian Data Protection Authority (NSD) 14/01407-5/EOL. The authors state that there are no conflicts of interest in either the study or the analysis presented in this manuscript.
